# Preharvest melatonin foliar treatments enhance postharvest longevity of cut tuberose *via* altering physio-biochemical traits

**DOI:** 10.3389/fpls.2023.1151722

**Published:** 2023-03-23

**Authors:** Faisal Zulfiqar, Anam Moosa, Anastasios Darras, Muhammad Nafees, Antonio Ferrante, Kadambot H. M. Siddique

**Affiliations:** ^1^ Department of Horticultural Sciences, Faculty of Agriculture and Environment, The Islamia University of Bahawalpur, Bahawalpur, Pakistan; ^2^ Department of Plant Pathology, Faculty of Agriculture and Environment, The Islamia University of Bahawalpur, Bahawalpur, Pakistan; ^3^ Department of Agriculture, University of the Peloponnese, Kalamata, Greece; ^4^ Department of Agricultural and Environmental Sciences, Università degli Studi di Milano, Milan, Italy; ^5^ The University of Western Australia (UWA) Institute of Agriculture, The University of Western Australia, Perth, WA, Australia

**Keywords:** oxidative stress, postharvest, ornamental cut flower, soluble proteins, soluble sugars, antioxidants

## Abstract

**Introduction:**

Melatonin (MLT) is a bioactive molecule involved in the physiological functioning of plants. Reports related to preharvest applications of melatonin on the postharvest performance of cut flowers are not available in the literature.

**Materials & methods:**

This study evaluated the effects of different concentrations of exogenous MLT [0 mM (MT0), 0.5 mM (MT1), 0.7 mM (MT2), 1 mM (MT3)] applied preharvest on the physiological characteristics and postharvest performance of cut tuberose, a globally demanded cut flower.

**Results & discussion:**

The results revealed that all treatments increased postharvest vase life by up to 4 d. The MT1, MT2, and MT3 treatments increased total soluble proteins (TSP) by 25%, 41%, and 17%, soluble sugars (SS) by 21%, 36%, and 33%, an+d postharvest catalase (CAT) activity by 52%, 66%, and 70%, respectively. Malondialdehyde (MDA) and hydrogen peroxide (H_2_O_2_) decreased in all preharvest treatments by up to 23% and 56%, respectively. Proline concentration decreased in all treatments, particularly MT3 (38%). These findings suggest that preharvest MLT treatment is a promising strategy for improving the postharvest quality of cut tuberose.

## Introduction

Tuberose (Asparagaceae) is a herbaceous perennial tropical and subtropical ornamental geophyte native to Mexico. Its tubular, sweet-scented white flowers are economically important as cut flowers, fragrance, and aromatic oil. The inflorescence of tuberose has a spike ranging from 90–120 cm, arranged in single or paired, waxy, highly fragrant flowers (Dole and Wilkins, 2005) that can be harvested for commercial purposes or used in landscape designs.

The vase life of cut flowers is a critical quality influencing profit margins for growers in national and international markets (Zulfiqar and Ashraf, 2022). After harvest, the metabolic activities of flowers remain active in cells, performing crucial processes using stored substrates in the tissues (Jhanji et al., 2023). Enhanced vase life and delayed senescence can be attained by maintaining carbohydrate levels and water absorption and ameliorating oxidative stress produced by excessive reactive oxygen species (ROS) generation (Zulfiqar and Ashraf, 2022).

Techniques for delaying the senescence of cut flowers can significantly increase their market potential since postharvest longevity is a crucial factor for cut flower value (Olsen et al., 2015). Various postharvest treatments using growth regulators, sugars, signaling molecules, and biostimulants can inhibit postharvest senescence and increase vase life (Zulfiqar et al., 2020). However, few studies have focused on preharvest applications of these substances to enhance the postharvest performance of cut flowers. Recently, preharvest applications of biostimulants and potassium enhanced postharvest performance of gladiolus and statice cut flowers by altering postharvest physiological conditions and mitigating oxidative stress during senescence (Zulfiqar et al., 2020; Khandan-Mirkohi et al., 2021; Zulfiqar and Ashraf, 2022).

Melatonin (MLT; N-acetyl-5-methoxytrytamine) is a multi-regulatory pleiotropic molecule involved in various physiological and cellular functions in response to biotic and abiotic stresses (Zhang et al., 2018; Arnao et al., 2022). Due to its antioxidant impact, MLT can prevent oxidative stress in plants (Altaf et al., 2021a; Altaf et al., 2021b; Altaf et al., 2022). Treatments with MLT extended the postharvest quality and shelf life of various horticultural products (Luo et al., 2020; Wang et al., 2020; Lin et al., 2022) by regulating gene expression and inducing antioxidant enzyme production (Zheng et al., 2019; Aghdam et al., 2021). However, MLT application for improving floricultural products is in its infancy. A recent study on cut carnations (*Dianthus caryophyllus* L) evaluated different MLT concentrations (0.01, 0.1, and 1 mM) added to the vase solution (Lezoul et al., 2022). The optimum concentration (0.1 mM) decreased senescence and increased vase life by up to 10 d compared to the untreated controls. The authors found that postharvest MLT treatments improved water relations, lowered metabolic rate, and maintained membrane stability due to antioxidant activity (Lezoul et al., 2022).

To date, no studies have investigated the effects of preharvest MLT applications on growth, vase life traits, and oxidative stress-related characteristics in tuberose plants. Therefore, we hypothesized that exogenous MLT improves postharvest performance and delays senescence in cut tuberose. We assessed the effect of different concentrations of foliar MLT applications on the growth and ornamental traits of tuberose plants and the association between photosynthesis and postharvest flower longevity and enzyme activities that reduce oxidative stress during senescence.

## Materials and methods

### Experimental site and plant material

An outdoor pot trial was established in the summer of 2022 at the Floriculture Research Area of the Islamia University of Bahawalpur, Pakistan (lat. 29° 23’ 44.5956’’ N, long.71° 41’ 0.0024” E). The desert region of Bahawalpur is in the subtropical zone, associated with hot summers (March–August) and mild winters (December–February). The physio-chemical traits of the experimental soil were: sandy clay loam (sand 45%, silt 24%, clay 31%), pH 7.4, 2.78 dSm^–1^ electrical conductivity, and 4.04 cmol_c_ kg^−1^ cation exchange capacity. Soil nutrients were: nitrogen (N), 79 g kg^–1^ soil; phosphorus (P), 9.03 g kg^–1^ soil; potassium (K), 152.54 g kg^–1^ soil. The soil was air-dried, ground, and sieved (2 mm pore size) ahead of filling 3 L earthen pots (19 cm and 13 cm top and base diameters, respectively).

Healthy, uniform tuberose bulbs of cv. Single (21–23 mm diameter) were acquired from a local supplier in Lahore, Pakistan. One tuberose bulb was planted per pot. There were ten replicate bulbs for each of the four treatments and four replications (total 160 plants). Basal N, P, and K fertilizers (6 g pot^–1^) were applied manually using 46% urea, 50% muriate of potash (Fauji Fertilizer Company Limited, Pakistan), and 18% single super phosphate (Safi Chemicals and Fertilizer (PVT) Limited, Multan, Pakistan). Second and third applications of these fertilizers at 25 d and 40 d after planting. The experiment had a completely randomized design with four treatments: (1) distilled water used as the control (MT0), (2) 0.05 mM MLT (MT1), (3) 0.07 mM MLT (MT2), and (4) 1 mM MLT (MT3). The treatments were applied in the middle of the growing cycle and 5 d before inflorescence harvest by manually spraying the leaves until run-off. Before each foliar spray application, the top of each pot was concealed with polyethylene sheeting to avoid contamination. Watering was done manually every four days until harvest.

### Leaf gas exchange

Leaf gas exchange traits [net CO_2_ assimilation (*A_s_*), and transpiration (*E*)] were measured at the onset of the flowering bud occurrence stage between 7.00 am and 8.00 am on three fully expanded leaf blades using an infrared gas analyzer (LI-COR 6400, LI-COR, Lincoln, NE, USA) at 400 μmol m^−2^ s^−1^ CO_2_ and flow rate of 300 μmol m^−2^ s^−1^ on eight plants per treatment. At the same time, chlorophyll SPAD values were recorded on the lower, middle, and tip parts of four fully expanded tuberose plant leaves.

### Harvest and vase life

At the initiation of the inflorescence maturity stage, uniform size and quality inflorescences were cut manually using a sterilized knife in the early morning (7:00 am to 8:00 am), placed vertically in a bucket half-filled with distilled water and transferred to the laboratory within 30 min. In laboratory, the inflorescences were re-cut at 85 cm length under running distilled water to revert vascular system blockage and air emboli. Individual inflorescences were placed into 200 mL glass vases containing deionized distilled water. The vases were covered with aluminum foil to reduce vase water evaporation and placed on laboratory bench at 26 ± 3°C, 65 ± 3% relative humidity, 12 h light period provided by white fluorescent lamps, and 12 h dark period. Ten cut inflorescences per treatment were kept for vase life determination. Vase life expiration was calculated as the number of days from harvest until the flower petals wilted and lost their visual aesthetic by color change and/or loss of turgidity. Data were documented daily for 15 d.

### Soluble sugar (SS) and total soluble protein (TSP) contents in leaves

The SS content (g kg^–1^) in 0.5 g tuberose leaves was measured 5 d before inflorescence harvest following the methodology of Frohlich and Kutscherah (1995), with absorbance measured at 620 nm. The TSP content (g kg^–1^; fresh weight basis) of tuberose leaves was determined following the methodology of Bradford (1976).

### Hydrogen peroxide (H_2_O_2_) and malondialdehyde (MDA) contents

Cut flower florets (0.5 g) of the inflorescence were taken on day 5 postharvest to assess H_2_O_2_ and MDA contents following the methodologies of Patterson et al. (1984) and Hodges et al. (1999), respectively.

### Superoxide dismutase (SOD) and catalase (CAT) activities

Fresh floret samples (0.5 g) were collected from the inflorescence on day 5 postharvest to assess SOD and CAT activities in stored supernatant following the methodologies of van Rossum et al. (1997) and Chance and Maehly (1955), respectively.

### Proline content

The ninhydrin-oriented method was used to measure leaf proline concentration (Bates et al., 1973), with absorbance read at 520 nm.

### Experimental lay-out and statistical analysis

Experiments were conducted in a CRD with MLT treatments as the only factor and were replicated four times. Data were subjected to one-way ANOVA using SPSS v. 21 (SPSS Inc., Chicago, IL, USA). Comparisons between treatment means were carried out using the LSD multiple range test at P = 0.05. Linear regression analysis (y = ax+b) was performed in Sigmaplot 10 (Systat software Inc. USA) to highlight the dynamic trends in MLT effectiveness.

## Results

### Leaf gas exchange and chlorophyll content

**Figure 1:**
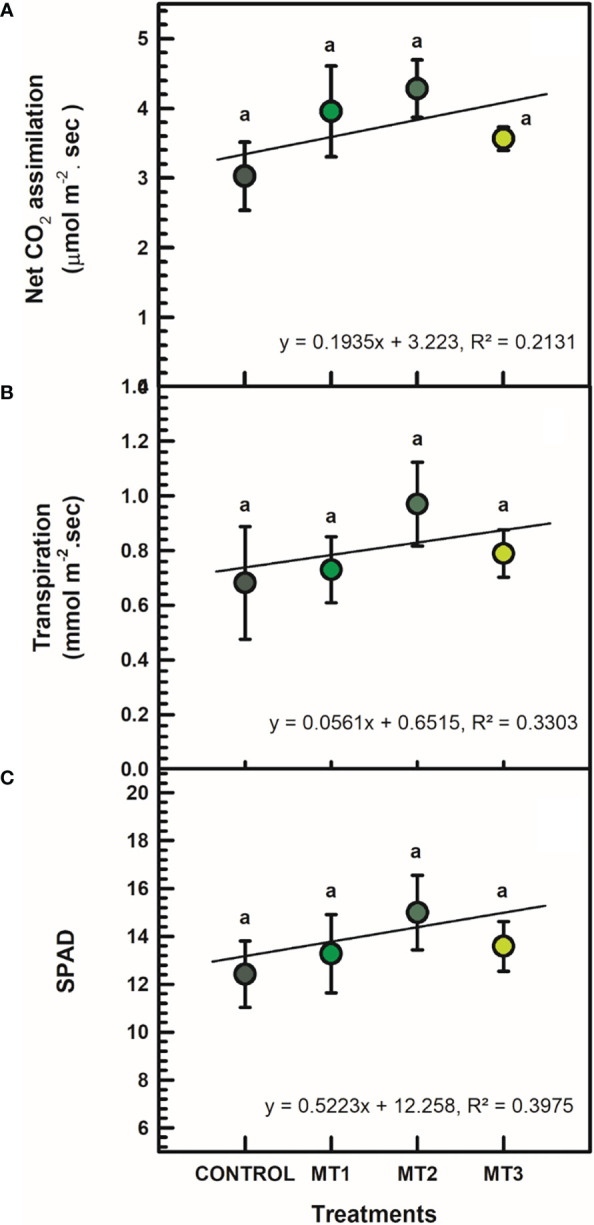
Net CO_2_ assimilation (**A**; μmol m^-2^ sec), transpiration (**B**; mmol m^-2^ sec), and SPAD **(C)** of tuberose plants treated with preharvest foliar applications of distilled water (control; MT0) and three concentrations of melatonin (MLT): 0.05 mM (MT1), 0.07 mM (MT2), and 1 mM (MT3). Data are means ± SE (n = 40). Different letters above the bars indicate significant differences according to the LSD test at P = 0.05.

MLT did not affect leaf gas exchange and chlorophyll content. All differences between treatment means were not significant at P = 0.05 (**Figure 1**). Slight increases in *As* and *E* values were recorded compared to the untreated control plants. For example, plants treated with MT2 had *As* value of 4.28 μmol m^-2^. sec, whereas the control plants had 3.03 μmol m^-2^. sec (**Figure 1A**). Likewise, E values of the MT2 treated plants averaged at 0.97 mmol m^-2^. sec, whereas the control plants averaged at 0.68 mmol m^-2^. sec (**Figure 1B**). SPAD values of the MT2 treated plants reached 14.99, whereas the untreated control plants 12.41 (**Figure 1C**).

### Vase life

**Figure 2:**
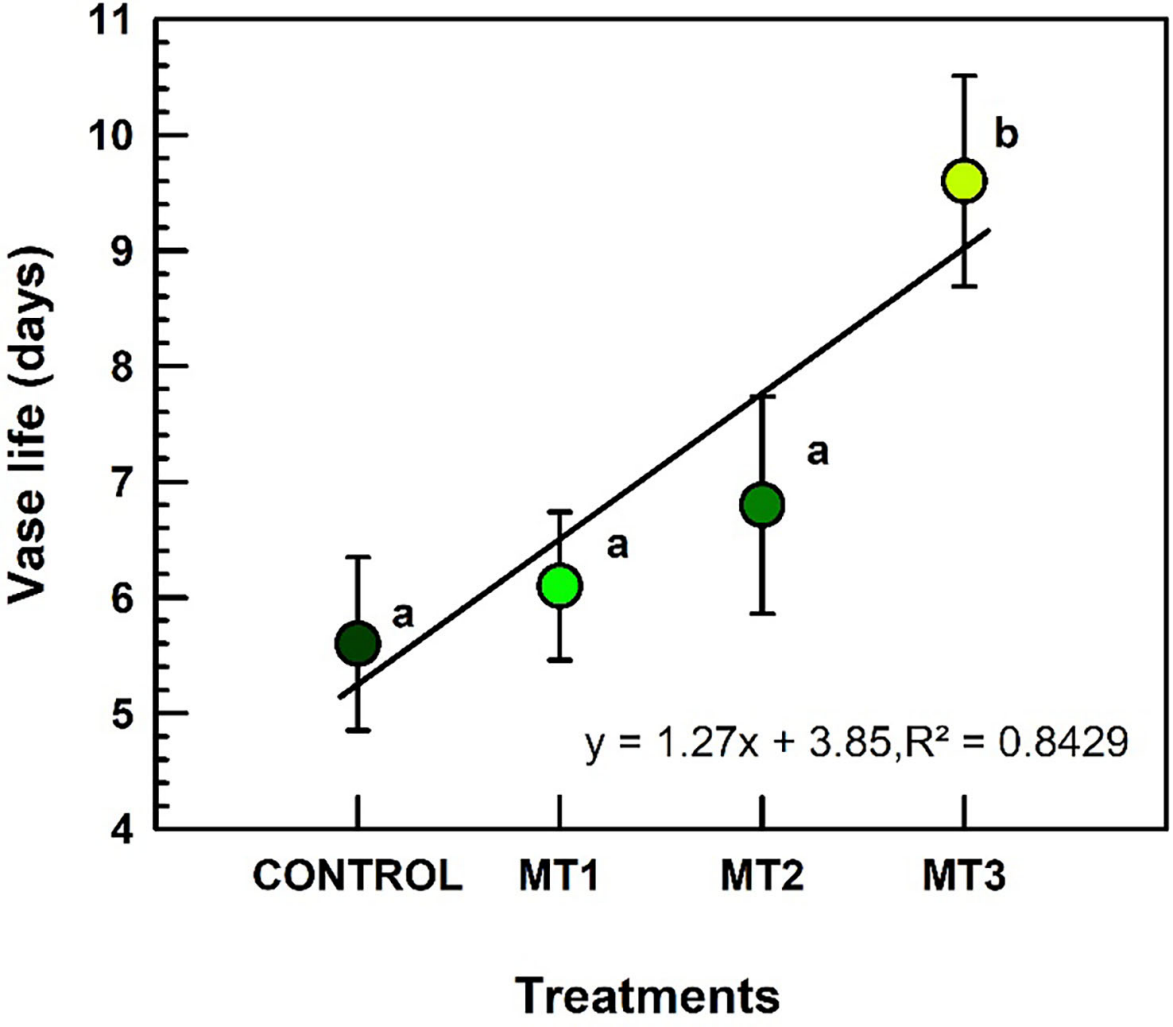
Vase life (d) of tuberose flowers harvested from the plants treated with preharvest foliar applications of distilled water (control; MT0) and three concentrations of melatonin (MLT): 0.05 mM (MT1), 0.07 mM (MT2), and 1 mM (MT3). Data are means ± SE (n = 40). Different letters above the bars indicate significant differences according to the LSD test at P = 0.05.

Vase life increased only in MT3 treated plants (**Figure 2**). Plants treated with MT3 produced inflorescences with the longest VL of 9.6 d (increase by 41%), compared to the 5.6 d recorded for the control inflorescences (**Figure 2**).

### Total soluble protein (TSP), soluble sugars (SS), and proline contents in leaves

**Figure 3:**
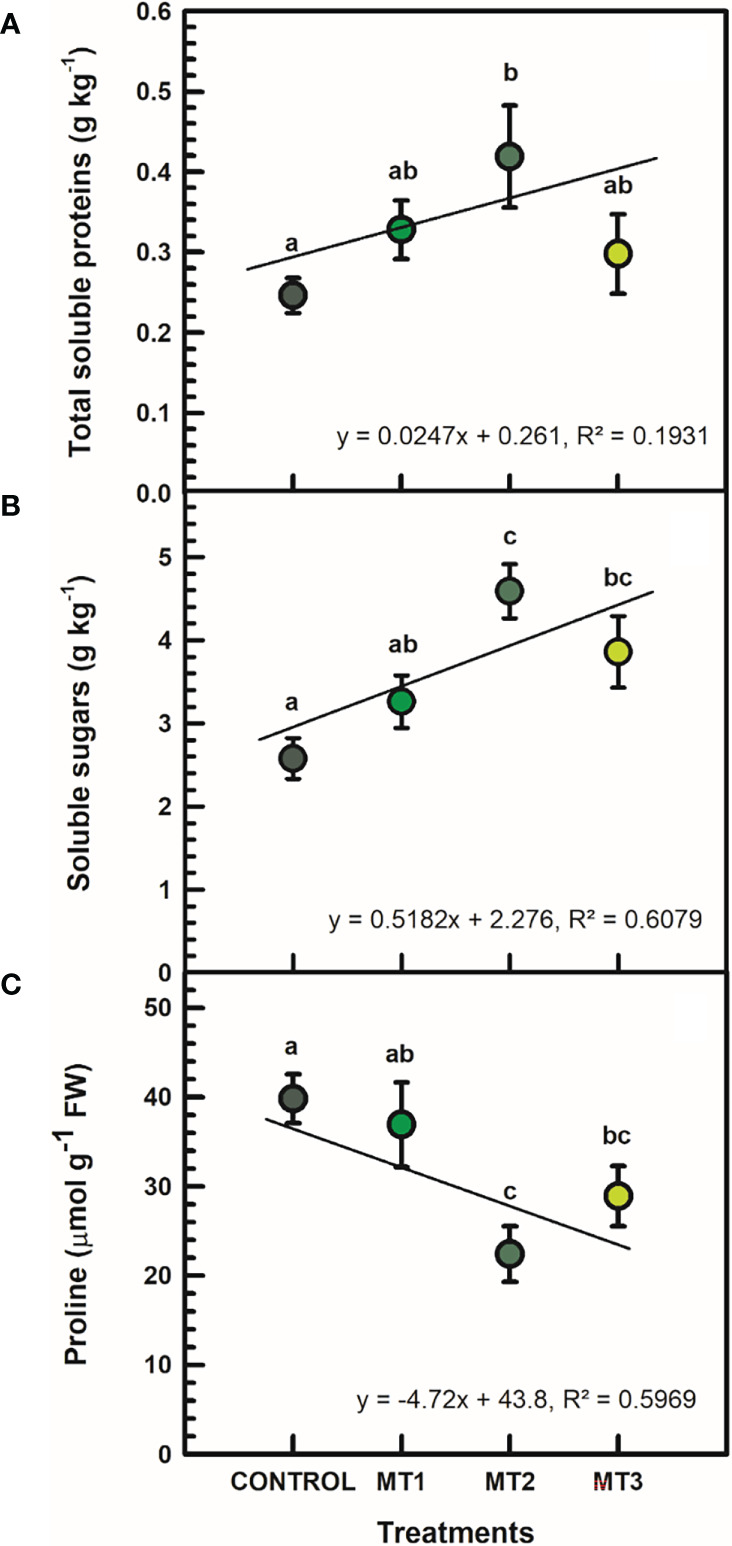
Total soluble protein (TSP) (**A**; g kg-1), soluble sugars (SS) (**B**; g kg-1), and proline contents (**C**; mmol g-1 FW) in leaves of tuberose plants treated with preharvest foliar applications of distilled water (control; MT0) and three concentrations of melatonin (MLT): 0.05 mM (MT1), 0.07 mM (MT2), and 1 mM (MT3). Data are means ± SE (n = 40). Different letters above the bars indicate significant differences according to the LSD test at P = 0.05.

Plants treated with MT2 showed a significant increase in TSP and SS (**Figures 3A, B**). MT2 treated plants had a TSP mean value of 0.42 g kg^-1^, whereas the untreated controls averaged at 0.33 g kg^-1^ (**Figure 3A**). That was an average increase of 41%. Likewise, MT2 treated plants had a SS mean value of 4.59 g kg^-1^, whereas the untreated controls averaged at 3.26 g kg^-1^ (**Figure 3B**). This increase was by up to 41% between the MT2-treated and the untreated plants. Additionally, the proline content in MT2-treated plants was significantly reduced by up to 78%, compared to the controls (**Figure 3C**). The MT2-treated plants had a proline content of 22.4 μmol g^-1^ FW, whereas the untreated control plants averaged at 39.8 μmol g^-1^ FW (**Figure 3C**).

### Hydrogen peroxide (H_2_O_2_) and malondialdehyde (MDA) contents

**Figure 4:**
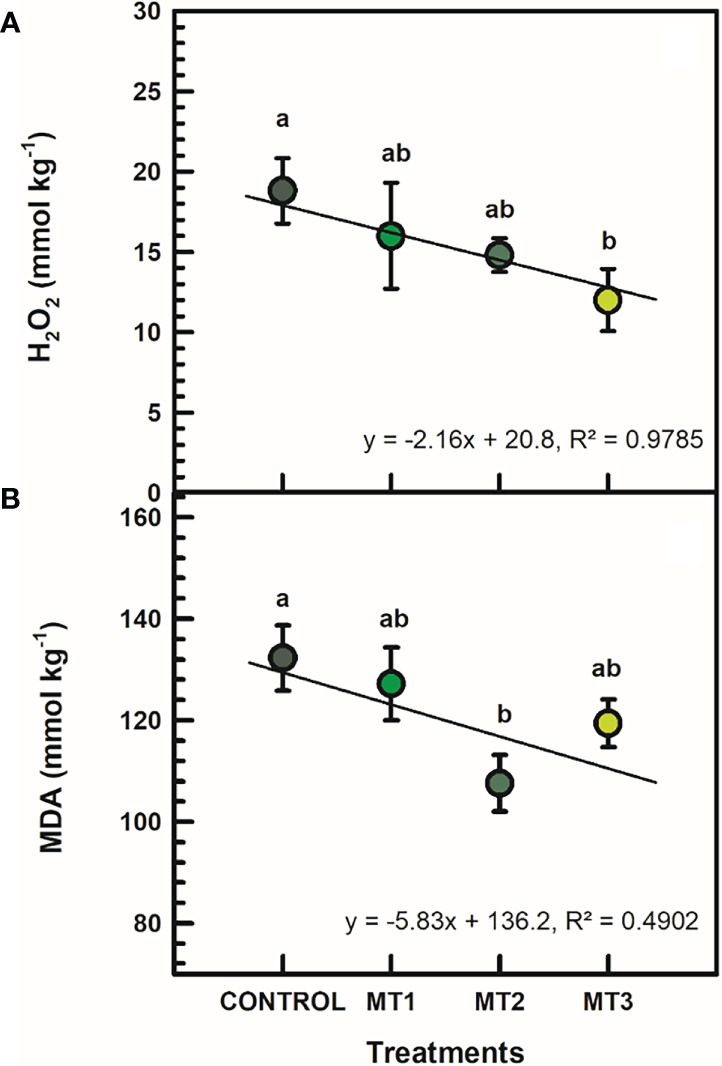
Hydrogen peroxide (H2O2) (**A**; mmol kg-1) and malondialdehyde (MDA) (**B**; mmol kg-1) contents in flowers of tuberose plants treated with preharvest foliar applications of distilled water (control; MT0) and three concentrations of melatonin (MLT): 0.05 mM (MT1), 0.07 mM (MT2), and 1 mM (MT3). Data are means ± SE (n = 40). Different letters above the bars indicate significant differences according to the LSD test at P = 0.05.

H_2_O_2_ and MDA contents were generally reduced by MLT treatments (**Figure 4**). H_2_O_2_ was significantly reduced in MT3 plants by up to 56% (**Figure 4A**). MT3-treated plants had a H_2_O_2_ content of 12 mmol kg^-1^, whereas the untreated control had 18.8 mmol kg^-1^. Furthermore, the MT2-treated plants showed a significantly reduced MDA content by 23% compared to the controls (**Figure 4B**). MT2-treated plants showed an average of 107.6 mmol kg^-1^ MDA, whereas the untreated control 132.3 mmol kg^-1^.

### Superoxide dismutase (SOD) and catalase (CAT) activities

**Figure 5:**
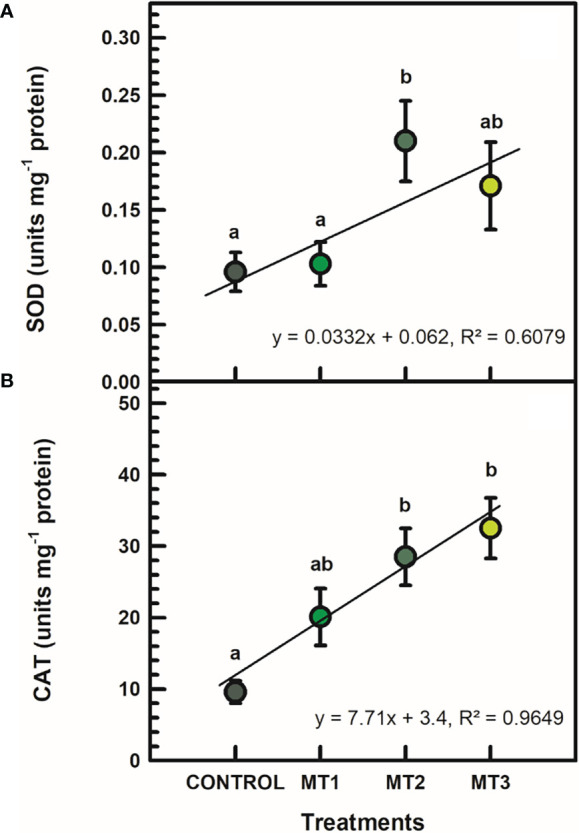
Superoxide dismutase (SOD) (**A**; units mg^-1^ protein) and catalase (CAT) (**B**; units mg^-1^ protein)activities in flowers of tuberose plants treated with preharvest foliar applications of distilled water (control; MT0) and three concentrations of melatonin (MLT): 0.05 mM (MT1), 0.07 mM (MT2), and 1 mM (MT3). Data are means ± SE (n = 40). Different letters above the bars indicate significant differences according to the LSD test at P = 0.05.

SOD and CAT activities were significantly induced by MLT only in certain cases (**Figure 5**). SOD was significantly increased by up to 54% in the MT2-treated plants (**Figure 5A**). SOD in MT2-treated plants was 0.21 units mg^-1^ protein and 0.01 units mg^-1^ protein in the untreated control plants (**Figure 5A**). CAT activity was increase by up to 70% in the MT3-treated plants (**Figure 5B**). CAT in MT3-treated plants was 32.5 units mg^-1^ protein and 9.6 units mg^-1^ protein in the untreated controls (**Figure 5B**).

## Discussion

Numerous studies have discovered that endogenous MLT content is involved in floral senescence. However, among different flower species, MLT levels appear to decline during development, particularly at later phases of senescence (Murch et al., 2009; Zhao et al., 2017). In the current study, preharvest MLT treatments enhanced physiological traits and had an anti-senescent effect on tuberose cut flowers, elongating vase life, particularly in MT3. The increased vase life was associated with improved biochemical characteristics, as the MLT treatments controlled antioxidant defenses postharvest for longer than the control.

Photosynthesis is the primary process for harnessing light energy to manufacture carbohydrates, and is closely linked to plant growth. The MLT treatments enhanced photosynthetic activity in tuberose, evident in the leaf gas exchange properties (**Figure 1A**), and in line with similar studies on cotton (Khattak et al., 2022) and tomato (Altaf et al., 2022). In contrast, Zhao et al. (2021) reported that MLT treatments did not affect leaf gas exchange in maize (*Zea mays*) under normal conditions. Furthermore, MLT has a protective impact on chlorophyll (Campos et al., 2019; Li et al., 2021; Altaf et al., 2022). The MLT-treated tuberose plants had more chlorophyll than control plants (**Figure 1C**), showing that exogenous MLT inhibits photosynthetic machinery damage. An increased photosynthetic capability provides plants with more energy, allowing them to withstand stressors like postharvest stress (Fan et al., 2015).

This study is the first to investigate the effect of preharvest MLT on the vase life of cut tuberose flowers. The prolonged vase life with preharvest MLT is likely related to enhanced photosynthesis, soluble sugars and antixodant defense system. In addition, the increased protein content and antioxidant activity with preharvest MLT may have reduced the oxidative damage in tuberose tissues, extending the vase life. At the highest dose (MT3), this cost-effective preharvest treatment could benefit cut flower sellers, prolonging tuberose vase life. Lezoul et al. (2022) reported that MLT improved the vase life of carnation due to its antioxidative potential. Our results showed improved vase life, which also shows that MLT mitigates oxidative stress, as reported by Mazrou et al. (2022).

The water balance in petals is crucial for extending the vase life of cut flowers. Numerous investigations have revealed a strong connection between water balance and the capacity of cut flowers for osmotic adjustment (Hou et al., 2018; Zheng and Guo, 2019; Lu et al., 2020). In plants, the levels of osmolytes, such as soluble proteins, soluble sugars, and proline, are closely linked with the ability to modify osmotic pressure. The data increasingly indicates that exogenous substances could improve water balance by controlling osmolyte concentrations (Shan and Zhao, 2015). Shan and Zhao (2015) demonstrated that lanthanum improved water balance in *Lilium longiflorum* cut flowers by increasing soluble protein, soluble sugar, and proline contents, further enhancing the relative water content of the petals and extending vase life. We found that MLT increased soluble protein and sugar contents in tuberose leaves, consistent with Xing et al. (2021) for chrysanthemum seedlings. Proline, an osmotic adjustment chemical, in addition to the cell’s antioxidant system, participates in the defense mechanism against adverse situations (Zulfiqar and Ashraf, 2022). Proline is a non-polar amino acid renowned for its numerous and significant roles in plant metabolism, particularly in response to biotic and abiotic stresses (Zulfiqar and Ashraf, 2022). Under stress, proline functions as a signaling molecule, compatible osmolyte, non-enzymatic antioxidant, molecular chaperone, and energy provider (Szepesi and Szőllősi, 2018). Studies have shown that proline content plays a vital role in the postharvest performance of cut flowers (Aghdam et al., 2019; Sukpitak and Seraypheap, 2023; Zeng et al., 2023). For example, sucrose application to rose cut flowers prolonged vase life, compared to non-treated cut flowers, which was associated with proline content (Zeng et al., 2023).

In the current study, the MLT treatments decreased MDA content, reflecting a decrease in lipid peroxidation in cut flowers, hence maintaining membrane integrity. Other studies have shown that MLT reduces lipid peroxidation while preserving the membrane stability index (Hassan et al., 2020; Zulfiqar and Ashraf, 2022).

Antioxidant enzymes such as SOD and CAT are the most important defense enzymes for ROS detoxification in plant tissues (Hasanuzzaman et al., 2020; Zulfiqar and Ashraf, 2021). The MLT treatments, especially at 0.07 and 1 mM, increased SOD and CAT activities in tuberose cut flowers. Horticultural commodities, including cut flowers, have increased antioxidant enzyme activity, reducing lipid peroxidation and H_2_O_2_ concentration during their postharvest (Lezoul et al., 2022; Mazrou et al., 2022). Several studies have demonstrated that MLT treatments increased antioxidant enzyme activities (e.g., SOD and CAT) in horticultural produce (Sharafi et al., 2021; Mazrou et al., 2022). In the current study, the improved antioxidant activities with MLT were related to decreased oxidative stress, as indicated by the reductions in H_2_O_2_ and MDA.

## Conclusion

The 0.07 and 1 mM preharvest MLT treatments were the most effective in delaying the senescence of tuberose cut flowers by improving leaf gas exchange, and TSP and soluble sugar contents and decreasing proline, H_2_O_2_, and MDA contents. Furthermore, these treatments increased SOD and CAT activities, decreasing oxidative stress. Preharvest MLT treatments at specific concentrations could prolong the vase life of tuberose cut inflorescences.

## Data availability statement

The raw data supporting the conclusions of this article will be made available by the authors, without undue reservation.

## Author contributions

FZ and AM designed and executed the experiments. All authors contributed on the writing, editing and revision of the manuscript. All authors contributed to the article and approved the submitted version.
